# Immunotherapy for type 1 diabetes mellitus by adjuvant-free *Schistosoma japonicum*-egg tip-loaded asymmetric microneedle patch (STAMP)

**DOI:** 10.1186/s12951-022-01581-9

**Published:** 2022-08-13

**Authors:** Haoming Huang, Dian Hu, Zhuo Chen, Jiarong Xu, Rengui Xu, Yusheng Gong, Zhengming Fang, Ting Wang, Wei Chen

**Affiliations:** 1 National Demonstration Center for Experimental Basic Medical Education, School of Basic Medicine, Tongji Medical College, Huazhong University of Science and Technology, Wuhan, Hubei 430030 China; 2 Department of Pharmacology, School of Basic Medicine, Tongji Medical College, Huazhong University of Science and Technology, Wuhan, Hubei 430030 China; 3 Department of Pathogen Biology, School of Basic Medicine, Tongji Medical College, Huazhong University of Science and Technology, Wuhan, Hubei 430030 China; 4 Hubei Key Laboratory for Drug Target Researches and Pharmacodynamic Evaluation, Huazhong University of Science and Technology, Wuhan, Hubei 430030 China

**Keywords:** Immunotherapy, Parasites-based therapy, Asymmetric microneedle, Autoimmune mediation, Type 1 diabetes

## Abstract

**Background:**

Type 1 diabetes mellitus (T1DM) is an autoimmune disease mediated by autoreactive T cells and dominated by Th1 response polarization. Insulin replacement therapy faces great challenges to this autoimmune disease, requiring highly frequent daily administration. Intriguingly, the progression of T1DM has proven to be prevented or attenuated by helminth infection or worm antigens for a relatively long term. However, the inevitable problems of low safety and poor compliance arise from infection with live worms or direct injection of antigens. Microneedles would be a promising candidate for local delivery of intact antigens, thus providing an opportunity for the clinical immunotherapy of parasitic products.

**Methods:**

We developed a *Schistosoma japonicum-*egg tip-loaded asymmetric microneedle patch (STAMP) system, which serves as a new strategy to combat TIDM. In order to improve retention time and reduce contamination risk, a specific imperfection was introduced on the STAMP (asymmetric structure), which allows the tip to quickly separate from the base layer, improving reaction time and patient’s comfort. After loading *Schistosoma japonicum-*egg as the immune regulator, the effects of STAMP on blood glucose control and pancreatic pathological progression improvement were evaluated in vivo. Meanwhile, the immunoregulatory mechanism and biosafety of STAMP were confirmed by histopathology, qRT-PCR, ELISA and Flow cytometric analysis.

**Results:**

Here, the newly developed STAMP was able to significantly reduce blood glucose and attenuate the pancreatic injury in T1DM mice independent of the adjuvants. The isolated *Schistosoma japonicum-*eggs micron slowly degraded in the skin and continuously released egg antigen for at least 2 weeks, ensuring localization and safety of antigen stimulation. This phenomenon should be attributed to the shift of Th2 immune response to reduce Th1 polarization.

**Conclusion:**

Our results exhibited that STAMP could significantly regulate the blood glucose level and attenuate pancreatic pathological injury in T1DM mice by balancing the Th1/Th2 immune responses, which is independent of adjuvants. This technology opens a new window for the application of parasite products in clinical immunotherapy.

**Supplementary Information:**

The online version contains supplementary material available at 10.1186/s12951-022-01581-9.

## Introduction

Diabetes is one of the most common endocrine and metabolic diseases, affecting around 537 million people nowadays, and this number may increase to 643 million by 2030 (11.3% of the global population) [1]. Among different categories, T1DM is mainly resulted from an autoimmune process, in which the body’s immune system attacks the insulin-producing β-cells of the pancreas, posing a great challenge to prevent the disease. Although it may develop at any age, T1DM mainly affects children and adolescents (1.2 million cases in 2021), accounting for approximate 90% of all types of childhood diabetes [2, 3]. In recent years, the incidence of T1DM has increased significantly by 2–5% worldwide, arousing urgent social and medical worry and concern to the whole population [2, 4]. Without prompt and effective treatment, T1DM patients may readily lose blood sugar control, leading to acute ketoacidosis and severe hypoglycemia, as well as complications including heart disease, kidney failure or blindness [5]. T1DM is believed to be a multifactorial consequence all along, however, there is overwhelming evidence that it is an autoimmune disease, typically mediated by an autoreactive T cell, characterized by a progressive immune destruction of insulin-producing β cells in the pancreatic islets, and subsequently resulting in absolute insulin deficiency and hyperglycemia within patients [5–8]. In this case, to date, insulin replacement therapy is the mainstay for T1DM treatment. Nevertheless, due to such inconvenience as lifelong insulin injection, poor compliance of young patients, long-term blood glucose monitoring and repeat health seeking, T1DM has brought a serious burden to the life, economy and emotion of patients and their family members [9, 10].

**Fig. 1:**
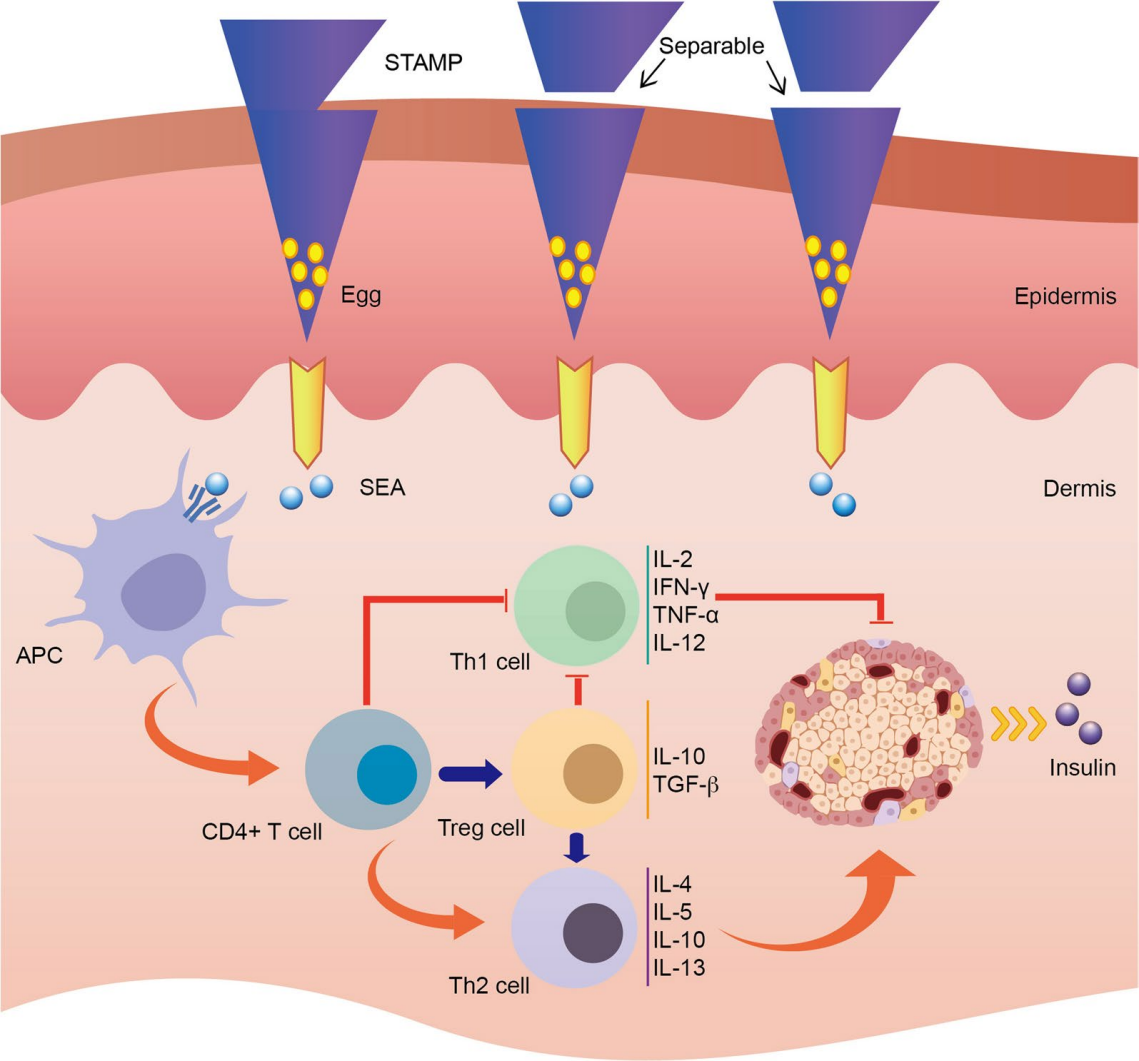
Immunological mechanism of STAMP application on the skin during the type 1 diabetes treatment. Schematic of STAMP delivering lyophilized *Schistosoma japonicum* eggs into the skin, and the reaction of dermal inflammation system and insulin secretion of pancreas islets. As the STAMP penetrated the stratum corneum by a thumb press, a shear force was applied to separate the tips off the base layer. The eggs remained in the epidermis after the biodegradation of CA-CMC and sustained the soluble egg antigen (SEA) release, interacting with local antigen-presenting cells (APC). Under the impact of antigen presented by APC, CD4^+^ T cells tended to differentiate into Th2 cells, which could produce protective cytokines (IL-4, IL-5, IL-10 and IL-13) to ameliorate the degree of pancreatic lesions and facilitate insulin secretion. Meanwhile, Th1 cell was down-regulated, leading to less secretion of the inflammatory cytokines (IL-2, IFN-γ, TNF-α and IL-12), thus reducing the damage of pancreatic β cells. Moreover, the regulatory Th cell (Treg cell) was up-regulated, generating more regulatory cytokines (IL-10 and TGF-β) to polarize the Th2 response and suppress the Th1 response

In the past few decades, numerous studies have been conducted to identify the causative factors of the T1DM with the aim of further discovering treatments or prevention in avoiding symptoms [11]. However, the etiology of T1DM has not yet been fully illustrated owing to multifactorial involvement [12]. On the basis of current knowledge, T1DM is considered to be an autoimmune disease resulting from a series of genetic, immunologic and environmental factors [12]. Self-antigens are processed by dendritic cells (DCs) and macrophages, presented in the form of antigenic peptide-loaded with major histocompatibility complex II (MHC-II) and then activate CD4^+^ T cells, which return to the islet β cells, recruit cytotoxic T cells and other inflammatory cells. IL-12 (Interleukin-12) secreted by activated DCs and macrophages stimulate the Th1 (T helper 1) cells releasing cytokines such as interferon-γ, IL-2 and TNF-α (tumor necrosis factor, TNF), etc., concurrently inhibit Th2 cells to secrete IL4, IL-5 and IL-10 etc. (Fig. 1), which further lead to cytotoxic T cells, macrophages and NKs (natural killer cell, NK) activation and ultimately islet β cells damage [5, 8]. The ensuing insulin deficiency and hyperglycemia require alternative exogenous insulin injections, which however cannot hold up the progression of autoimmune response and the life expectancy shortening of T1DM patients with complications [13, 14]. Therefore, this has greatly inspired scientists and doctors to develop various technical methods for etiological prevention or treatment of the T1DM, especially in aspects of immune therapy [15–17].

Notably, a variety of immunotherapies have been applied in clinical studies for T1DM therapy and brought great hope to the patients, such as immune-suppressants, immune-modulators, induction of immune tolerance and immunological intervention targeting genes [18, 19]. Yet so, facing the heterogeneity of type 1 diabetes, diversity of islet antigens and complexity of autoimmune processes, sufficient success has rarely been achieved to completely halt or reverse the progression of type 1 diabetes, and meanwhile, its safety, high price, ethical issues and long-term efficacy remain controversial [20–23]. The “hygiene hypothesis” proposed that improvement of sanitation and infrequent exposure of children to infections are the main triggers for the rise in autoimmune disorders, which was notable in the most significant increase in T1DM incidence in industrialized societies with decreasing exposure to parasites [24, 25]. Numerous evidence supported the potential efficacy of helminth infections and helminth derivatives in treating the T1DM mouse model [26–30]. *Schistosomiasis* infection and exposure to *schistosoma*-derived antigens have been shown to prevent Th1-mediated autoimmune diseases including T1DM, multiplesclerosis (MS) and Crohn’s disease [31, 32]. It was reported that *Schistosoma mansoni* infection or its adult/egg antigens are able to decrease the incidence of T1DM in rodents, and similar results were investigated in a broad spectrum of studies in other T1DM animal models with *Schistosoma japonicum* infection [31, 33–35]. T1DM has proven to be an autoimmune disease mainly mediated by Th1 cells, while *Schistosoma spp*. infection leads to the polarization of Th2 type immune response, thus weakening the activity of Th1 cells and arresting the occurrence or development of T1DM [36, 37]. As a co-evolving pathogen with humans, parasites regulate host immune responses and build up an anti-inflammatory microenvironment, which might provide a new strategy for the prevention and treatment of T1DM.

Most studies have been limited to living worm infection or crude worm proteins obtained from animal models, and the use of purified derivatives or proteins may not necessarily produce immunomodulatory effects similar to those induced by intact worm antigens in the host, and may not achieve desirable effects [34, 35, 38]. At the same time, the inevitable side effects caused by worm proteins, the safety of living worm infection and the rejection of patients make it difficult to accept parasites ethically. Hence parasite products are not considered for clinical application. To address the challenge of the immunotherapy of parasite-related products in T1DM, based on our previous success in microneedle-based strategies [39–43], herein we develop an asymmetric microneedle patch for the continuous and safe release of inactivated *Schistosoma japonicum* eggs. Microneedles are micron-sized needle-shaped structures that can penetrate the stratum corneum of the skin with a thumb press or an applicator to deliver drugs in a minimally invasive and painless way [44], which would significantly improve patient’s compliance. Conventional microneedle usually remains the patch base on the skin surface for a long time, triggering severe discomfort to the patient and posing a high infection risk [45–47]. Moreover, aiming to continuously mediate the immune-response of the host, the microneedles are required to sustained release the payload locally, allowing more chance for the released active components to interact with local dendritic cells and antigen-presenting cells [48, 49].

Given these challenges, we developed a *Schistosoma japonicum* egg tip-loaded asymmetric microneedle (STAMP), in which the base layer was designed as an easy-fracture structure (an imperfection on one side) and fixed only within the epidermis layers of the skin. The isolated microneedles were then biodegraded slowly in the skin with sustained release and topical delivery of encapsulated eggs immobilized in the epidermal layer of the skin for at least 2 weeks to well regulate the releasing amount of eggs and ensure the localization and safety of antigen stimulation. Intriguingly, the results indicated that the STAMP could effectively control blood glucose level and ameliorate the degree of pancreatic lesions in T1DM mice. Notably, different from other immunotherapy, the adjuvant seems to be not necessary in this system, possibly ascribing to the fact that the intact egg contains sufficient immunomodulatory components, simplifying the formulation preparation. Based on the results, we believe that the STAMP has exhibited the promising potential to serve as an efficient strategy to combat T1DM by providing long-lasting effects to prevent islet destruction of patients and reduce the occurrence of complications. This technique opens a new window in immunotherapy and may be utilized in other autoimmune diseases in the near future.

## Results

**Fig. 2:**
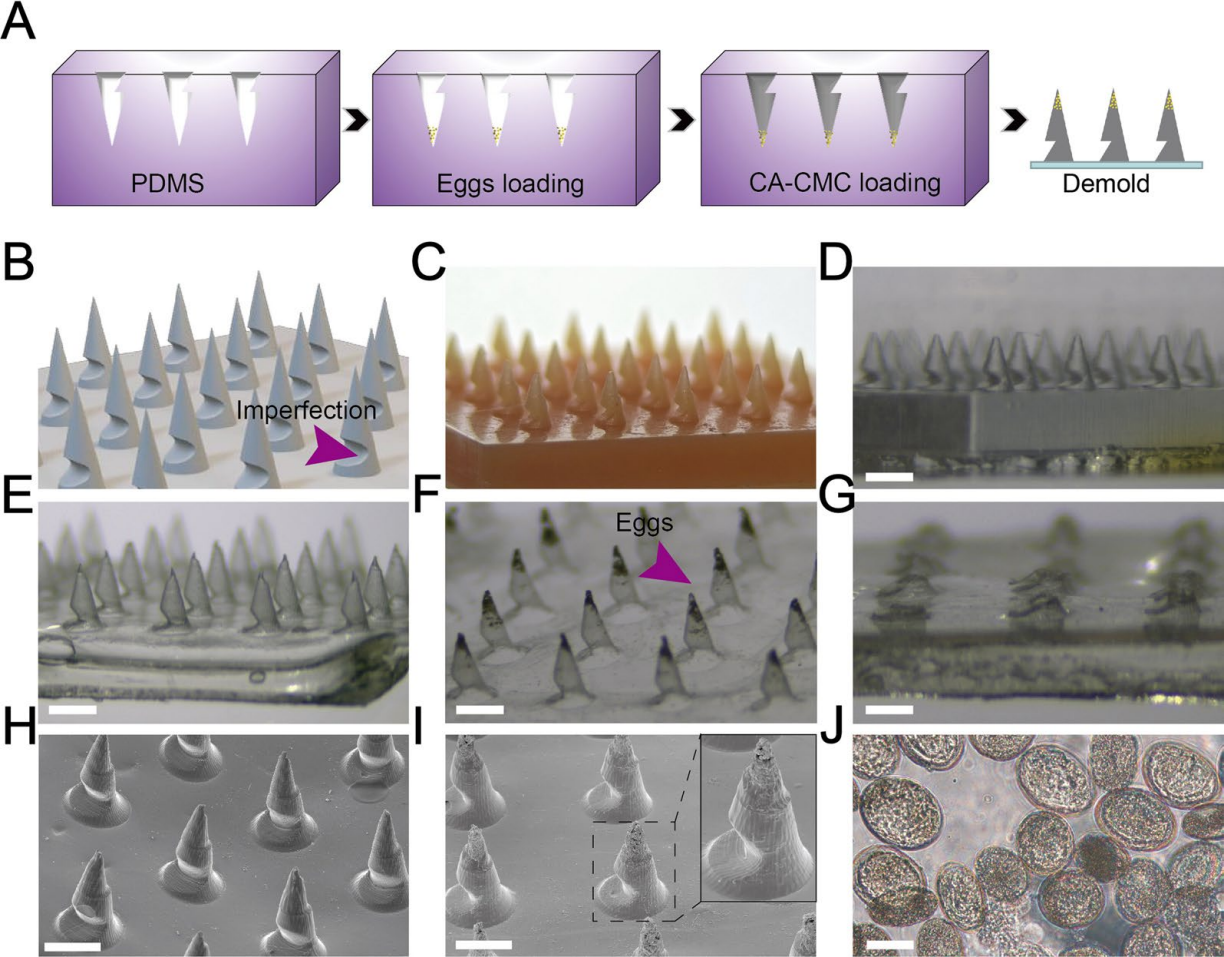
Fabrication and characterization of STAMP and blank microneedle patch. **A** Schematic diagram of manufacture process with a PDMS mold. **B** Designed model of the STAMP. The purple arrow indicated the imperfection. **C** Macroscopic view of the master mold. **D** Image of the PDMS mold under the stereomicroscope. Scale bar, 1 mm. **E** Stereomicroscopic image of the blank CA-CMC microneedle patch. Scale bar, 1 mm. **F** Stereomicroscopic image of STAMP with lyophilized *Schistosoma japonicum* eggs, which were obviously located on the tip as indicated by the purple arrow. Scale bar, 1 mm. **G** Stereomicroscopic image of a STAMP after penetration and tip-base separation. Scale bar, 1 mm. **H** SEM image of the blank CA-CMC microneedle patch. Scale bar, 1 mm. **I** SEM image of STAMP. Notably, the eggs were loaded in the tip. Scale bar, 1 mm. **J** Microscopic image of purified *Schistosoma japonicum* eggs. Scale bar, 100 μm

### Fabrication and characterization of the STAMP

When developing STAMP in a mold casting method (Fig. 2A), CA-CMC was selected as the supporting material because of its biosafety, sustainable degradation and sufficient mechanical strength. According to previous studies [50, 51], CMC-based material did not cause dermal toxicity and irritation, ensuring negligible inflammatory and allergy at the application site of the microneedles. As shown in Fig. 2B, we designed 5 × 5 array of conical tips with a total length of 0.9 mm and a diameter of 0.5 mm, each array contained 25 microneedles. However, distinguished from the traditional microneedles, on the junction of each tip and the base, there was an imperfection structure which allowed quick separation. It should be noted that the conventional microneedle without the imperfection was rigid, demanding larger shear to be deformed, thus might lead to more pain and destruction to the skin, which may result in patch loss without adequate active component release (Additional file 1: Fig. S1). After being manufactured according to the design, master mold (Fig. 2C) and PDMS mold (Fig. 2D) were observed under the stereoscopic microscope, indicating an evident imperfection structure. Blank CA-CMC microneedles used as a control in this experiment were simply made by CA-CMC casting, drying and demolding. The stereoscopic microscope image of blank microneedles (Fig. 2E) showed parallel tips distributed on the base surface with consistent imperfection on one side. In comparison, after loading with lyophilized *Schistosoma japonicum* eggs, the STAMP displayed a dark tip, indicating successful egg encapsulation as shown under a stereoscopic microscope (Fig. 2F). As expected, when the STAMP was applied on the pig skin, the tips were separated from the base, leaving a neat cut (Fig. 2G). Furthermore, the microstructure could be observed clearly in the blank CA-CMC microneedle patch (Fig. 2H) and the STAMP (Fig. 2I) under the SEM, while only STAMP displayed a rough tip, ascribing to the *Schistosoma japonicum* eggs (Fig. 2J) integration.

**Fig. 3:**
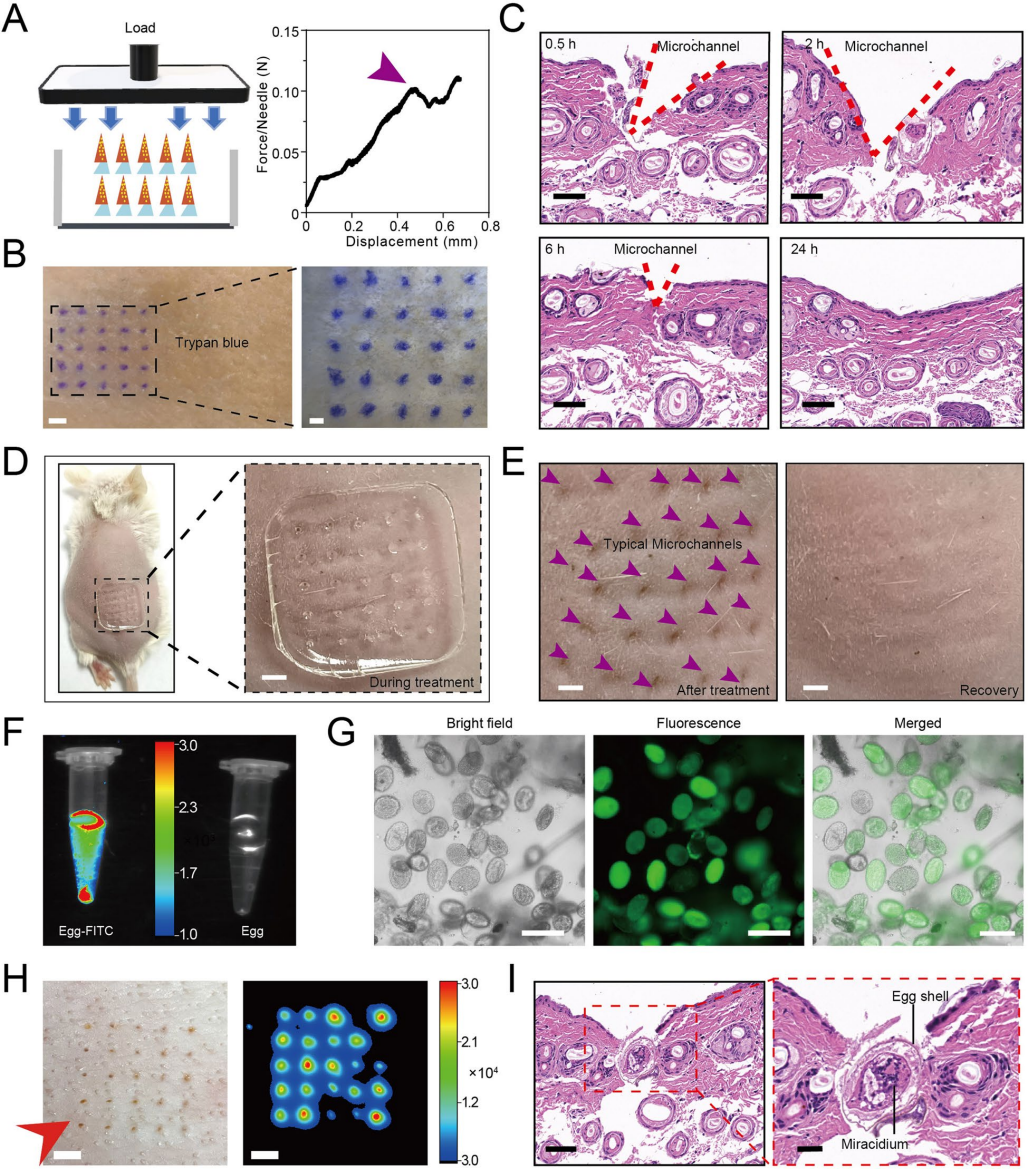
Penetration test of STAMP. **A** Schematic diagram of mechanical strength test of STAMP. The tips could be separated when the applied force is above 0.1 N (purple arrow). **B** Photography of STAMP, loaded with Trypan blue dye, inserted into the porcine skin. Typical microchannels could be investigated obviously under the microscope. Left scale bar, 2 mm; Right scale bar, 1 mm. **C** Pathological section of BALB/c mouse skin after microneedle insertion at different time points. Scale bar, 50 μm. **D** Photograph of BALB/c mouse skin during treatment. Scale bar, 1 mm. **E** Photograph of BALB/c mouse skin right after the treatment (left) and the recovered skin (right). Scale bar, 1 mm. **F** Fluorescence image of the FITC marked egg and the unmarked egg. **G** The bright-field, fluorescence and merged images of *Schistosoma japonicum* egg labeled by FITC, Scale bar, 200 μm. **H** The fluorescence image of porcine skin imprinted by STAMP, Scale bar, 1 mm. **I** The two sections below showed egg structures (the egg shell outside and the miracidium inside) at the skin incision, Scale bar, 50 μm

### Mechanism study and application of the STAMP

To assess if the STAMP system was able to penetrate the skin to deliver the payload, a failure force was tested (Fig. 3A). The force-displacement graph of STAMP demonstrated that STAMP was capable to tolerate compressive forces of over 0.10 N per needle, which was expected to enable skin puncture without breaking [52]. Compared with the conventional microneedles patch, the STAMP was readily to break, indicating effortless skin retention (Additional file 1: Fig. S2). To verify the penetration and payload release, the asymmetric microneedles loaded with Trypan Blue dye were inserted into porcine skin. After the administration, the blue dotted pattern (Trypan Blue dye) was remarkably visualized by naked eyes, suggesting successful intradermal delivery (Fig. 3B). In hematoxylin-eosin staining (HE staining) slides (Fig. 3C), the V-shaped microchannel was observed, which would spontaneously recover over time, indicating a non-invasive treatment. On the mouse skin, evident microchannels were observed after STAMP application, which would completely recover within 24 h (Fig. 3D, E). Additionally, we could observe the microneedles dissolving gradually within 24 h in the sepharose gel, which was similar to the endoepidermal environment (Additional file 1: Fig. S3). Based on the phenomenon, it is easy to conclude that STAMP would serve as a promising system to deliver parasitic products locally in a safe way with minimal damage. Besides, the eggs labeled by FITC were observed under the fluorescence microscope. In comparison with the original eggs with no autofluorescence (Additional file 1: Fig. S4), the FITC labeled eggs presented an obvious fluorescent dotted array on the porcine after STAMP delivery (Fig. 3F–H). Moreover, during the administration of STAMP, lyophilized *Schistosoma japonicum* eggs with intact structure (the miracidium and egg shell) was detected, further confirming payload release (Fig. 3I).

**Fig. 4:**
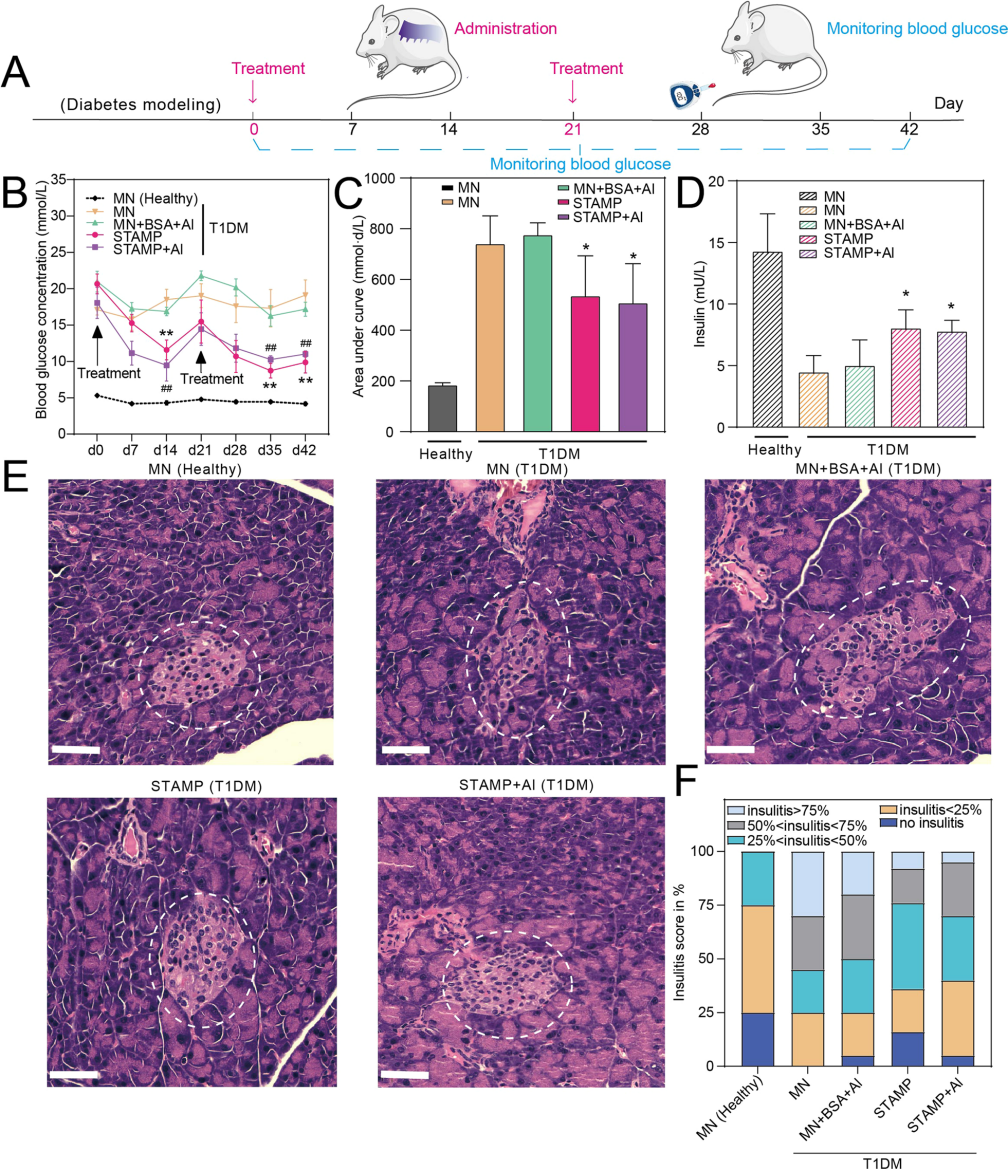
In vivo studies of STAMP for the T1DM treatment. **A** Schematic of the in vivo study operations. The arrows marked in red are the time points of the STAMP treatments. Blood glucose levels are measured every week from d0 after diabetes model establishment **B** Blood glucose concentration vs. time curves of different treatments. Data were presented as mean ± S.D. (n = 6). **p < 0.01, ^##^*p* < 0.01, compared with the mice in the MN T1DM group (two-tailed Student’s *t*-test). **C** Area under curve (AUC) analysis of blood glucose-time curves. Data were presented as mean ± S.D. (n = 6). *p < 0.05, compared with the data in the MN T1DM group (two-tailed Student’s *t*-test). **D** Insulin concentration in serum measured by ELISA. Data were presented as mean ± S.D. (n = 3). *p < 0.05, compared with the mice in the MN T1DM group (two-tailed Student’s *t*-test). **E** Pathological sections of mice pancreas in each group, pancreas islets identified by the dashed circles. Scale bar, 50 μm. **F** Insulitis scores of pancreas islets in 5 groups, according to the area proportion of inflammatory cells infiltrating into the islets

### In vivo studies of the STAMP for T1DM therapy

To evaluate the in vivo antidiabetic effect of STAMP for T1DM therapy, BALB/c mice were grouped and exposed transcutaneously to different administrations, including blank MN, MN + BSA + Al, STAMP and STAMP + Al. The amount of *Schistosoma japonicum* eggs or BSA in each group was set to around 500 µg after dose optimization (Additional file 1: Fig. S5). The blood glucose level (BGL) in each group was monitored on d0, d7, d14, d21, d28, d35 and d42 (Fig. 4A, B). Obviously, the BGL of T1DM mice was significantly higher than that of healthy mice, indicating successful model establishment. After the first administration, the BGL of the STAMP with or without Al groups continued to decrease (*p* < 0.01) within 14 days, compared with MN and MN + BSA + Al groups, suggesting effective BGL control by the eggs. Notably, the BGL of treated mice increased on the 21st day partly, thus the second administration was applied. Impressively, STAMP and STAMP + Al displayed a quick response again to regulate the BGL. It should be mentioned that the adjuvant Al seems to be not necessary in this system, possibly attributed to the fact that the intact eggs contain sufficient immunomodulatory components to mediate the whole immune process [53, 54]. In consistence, the BGL AUC of the STAMP and STAMP + Al groups was relatively lower (*p* < 0.05) compared with blank MN and MN + BSA + Al groups (Fig. 4B, C). The insulin level in serum was analyzed with enzyme-linked immunosorbent assay (ELISA), which indicated the inherent functions of the pancreas. Compared with the blank MN group, the blood insulin concentration was remarkably higher (*p* < 0.05) in the STAMP T1DM and STAMP + Al groups (Fig. 4D), corresponding to the BGL test and suggesting the protective effects of the eggs on the pancreases. Histological analysis of pancreatic islet inflammation showed a similar trend that STAMP exhibited better performance over others independent with adjuvant by displaying moderate insulitis (Fig. 4E, F). Collectively, all the results revealed that the STAMP could act as a promising candidate to combat T1DM in the mouse model by alleviating insulitis levels and improving insulin secretion. After the STAMP treatments, the body weight (Additional file 1: Fig. S6) and biochemistry markers (the alanine aminotransferase, aspartate aminotransferase, creatinine, leukocyte count, erythrocyte count and platelet count levels, Additional file 1: Fig. S7) were tested with no significant difference compared with untreated T1DM mice. Meanwhile, histological analysis of heart, liver, spleen, lung and kidney in all groups revealed no significant damage (Additional file 1: Fig. S8), suggesting a highly safe administration.

**Fig. 5:**
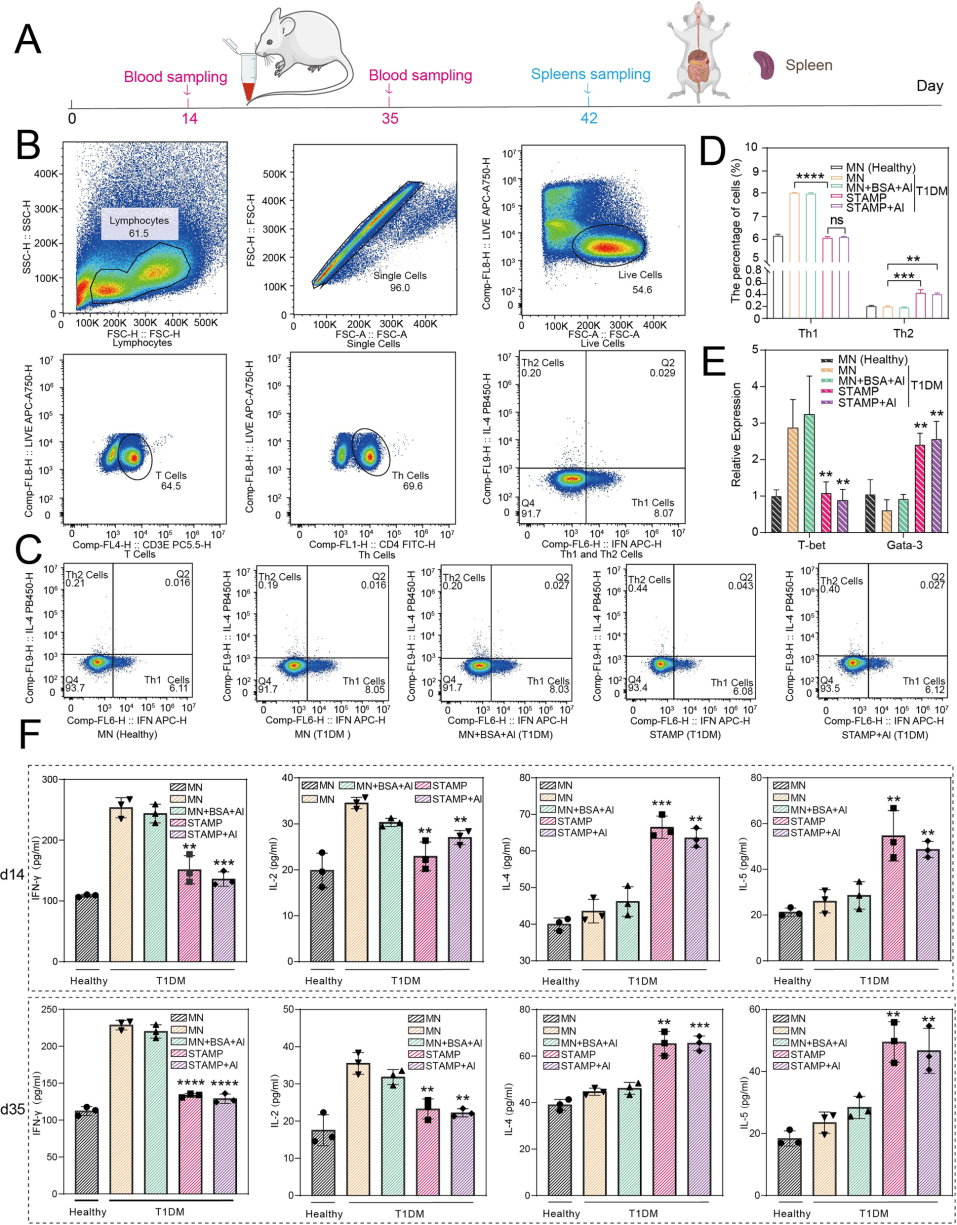
Flow cytometry, qRT-PCR and ELISA analysis for immune response in each group. **A** Schematic diagram of blood (red) and spleen (blue) collection. **B** Gating strategy to identify lymphocytes, single cells, live cells, T cells (CD3^+^), Th cells (CD3^+^CD4^+^), Th1 cells (CD3^+^CD4^+^IFN-γ^+^), and Th2 cells (CD3^+^CD4^+^IL-4^+^) in flow cytometry test. **C** Representative flow cytometry plots, showing the percentage of Th1 cells and Th2 cells among splenocytes of the five groups. **D** Statical analysis of fluorescence activating cell sorter (FACS) results. Results were shown as mean ± S.D. and represented three separate experiments (n = 6). **p < 0.01, ***p < 0.001, ****p < 0.0001, ns means no significant. **E** T-bet and Gata-3 mRNA expression examined by qRT-PCR using splenocytes collected on d42 after the first treatment. Data were presented as mean ± S.D. (n = 3) **p < 0.01, ***p < 0.001, ****p < 0.0001, ns meant no significance, compared with the data in the MN T1DM group. **F** Cytokine concentration of Th1 (IFN-γ and IL-2) and Th2 (IL-4 and IL-5) detected by ELISA. The first row showed the cytokine levels on d14, while the second row was the cytokine levels on d35. Data were presented as mean ± S.D. (n = 3). **p < 0.01, ***p < 0.001, ****p < 0.0001, compared with the mice in the MN T1DM group (two-tailed Student’s *t*-test)

### The STAMP regulated the Th1/Th2 immune response in T1DM mice

To explore the mechanism of T1DM treatment by the STAMP, the spleens were gained on d42 (Fig. 5A), then the proportion of Th1 cells and Th2 cells in splenocytes in each group were analyzed by flow cytometry. Compared with the MN T1DM group, IFN-γ expression in CD4^+^ T cells was remarkably reduced (*p* < 0.0001), but IL-4 expression in CD4^+^ T cells was significantly increased (*p* < 0.001) in the STAMP-treated T1DM mice, indicating Th1 to Th2 response shift. Notably, the MN + BSA + Al T1DM group would not provide much help, verifying that the immune response was mainly mediated by the delivered *Schistosoma japonicum* eggs. Moreover, the difference between the STAMP and STAMP + Al group was ignorable, certifying that the adjuvant was not indispensable in the system (Fig. 5B−D), consistent with blood glucose detection. Based on the observation, it is believed that the STAMP combated T1DM mainly by balancing the Th1/Th2 response. Specifically, the STAMP effectively inhibited the Th1 cells to release cytokines such as IFN-γ and concurrently stimulate Th2 cells secreting IL-4, providing a well regulatory profile.

### The STAMP adjusted the expression of Th1/Th2-related transcription factors and cytokine levels

To assess the level of Th1/Th2-related transcription factors, the splenocytes were collected on d42 (Fig. 5A), and then the expression of GATA-3 and T-bet that were involved in Th1/Th2 response was examined by semi-quantitative RT-PCR in splenocytes. Compared with the MN and MN + BSA + Al groups, the T-bet expression was decreased (*p* < 0.01) effectively in STAMP groups (with or without Al) while GATA-3 levels were apparently increased (*p* < 0.01) (Fig. 5E). Meanwhile, the serum was harvested on d14 and d35 (Fig. 5A) and the IFN-γ, IL-2, IL-4 and IL-5 levels in serum were analyzed by ELISA kits. As expected, the levels of the Th1 cytokines IFN-γ and IL-2 were significantly down-regulated (*p* < 0.01), but the levels of the Th2 cytokines IL-4 and IL-5 were relatively up-regulated (*p* < 0.01) after STAMP treatments (with or without Al) in comparison with other groups, no matter in first or second administration, suggesting the alteration triggered by the STAMP were timely and durable (Fig. 5F). Moreover, the cytokines concentration of the serum on d21 measured by ELISA showed that the levels of the Th1 cytokines IFN-γ and IL-2 rose up, while the levels of the Th2 cytokines IL-4 and IL-5 fell down in contrast to the levels on d14, which showed consistent changes in BGL (Additional file 1: Fig. S9), and suggested the imbalance between Th1 and Th2 immune response was strongly associated with the changes in BGL.

## Discussion

Previous epidemiological evidence had demonstrated a negative association between helminths infection and autoimmune or allergic diseases, including asthma, arthritis, T1DM, multiple sclerosis, and inflammatory bowel disease (IBD), which was postulated as the so-called “hygiene hypothesis” [55–57]. The vertebrate immune system coevolved under constant attack from the parasites, resulting in a balance between the host’s protective inflammatory mechanisms which control worm infection and the immune regulation caused by the parasite. During the last decades, studies on the mechanisms of immune regulation induced by parasitic worms may contribute to the development of new treatment strategies for inflammatory diseases such as inflammatory bowel disease, non-obese diabetes (NOD), collagen-induced arthritis and so on [25, 58]. A large amount of relevant studies involving experimental infection with helminth or treatment with parasite-derived components have proven to work wonders in practical applications of diverse animal experimental models or human experience, such as ingesting infectious *Trichuris suis* eggs for IBD, using filarial cystatin or products of *Ancylostoma ceylanicum* against collagen-induced arthritis, or taking hookworm protein for asthma therapy [59–62].

Non-sensitized CD4^+^ T cells (Th0) express different transcription factors under the induction of various cytokines, and then differentiate into effector T cells (Th1, Th2, and Th17) and regulatory T cells (Treg) with distinct functions and phenotypes [63]. Activated CD4^+^ T cells up-regulate IFN-γ expression in the presence of IL-12 through signal and activator of transcription 4 (STAT-4). Thus, stat-1 signaling mediated by IFN-γ activates Th1-type cell-specific transcription factors (T-bet), leading to Th1-oriented differentiation. While in the presence of IL-4, CD4^+^ T cells activate STAT-6 to induce Th2 cell-specific transcription factor (GATA-3), which differentiates toward Th2 response. Th1, Th2, Th17 and Treg are interlinked by various cytokines. IFN-γ and IL-2 promote Th1 but inhibit Th2 cell differentiation, and IL-4 and IL-5 promote Th2 but inhibit Th1 cell differentiation. These cells constitute a complex cytokine regulatory network, which not only ensures the normal immune response function of the body, but also prevents the body from pathological damage caused by excessive immune response [64]. The etiology of most autoimmune disorders is speculated to associate with an over the polarization of Th1 immune response, leading to an imbalance of the Th1/Th2 immune ratio [65]. Intriguingly, a highly conserved feature of helminth infection is the polarization of Th2 response and counter-regulation of Th1 response [66, 67]. Therefore, it is foreseeable for T1DM (a typical autoimmune disease) etiological treatment through the parasite-conducted modulatory immune pathway from the pro-inflammatory to anti-inflammatory transformation. Previous studies have verified that the incidence and progression of T1DM could be inhibited by parasite infection, or treatment with soluble egg antigen (SEA), or soluble worm antigen (SWA) of *Schistosoma mansoni* and *Schistosoma japonicum* [33, 35, 68–70]. The possible mechanisms of protection against T1DM involve the conversion from Th1 to Th2 response, the activation of Treg (CD4^+^CD25^+^FoxP3^+^ T cells) and Breg (CD5^+^CD19^+^B cells), the decrease of IFN-γ and IL-2, and the promotion of IL-4, IL-5, IL-10, and TGF-β, all of which are capable of inhibiting T1DM pathology [35, 69, 71, 72]. Although our previous study demonstrated that schistosome eggs were beneficial for T1DM [73], it is worth noting that schistosomiasis (infected with live worms) can result in a variety of pathological lesions in the liver and colon, and schistosome-based therapy may not be readily acceptable by patients ethically and psychologically [74]. Recently, certain monomolecules from parasites capable of down-regulating immune response have been isolated and considered as safe alternatives to live worm infection. However, these monomolecular derivatives or DC exosomes treated with SEA were not well effective in reducing the degree of inflammatory disease in mice compared to eggs or mix-antigen itself [35, 75, 76]. These findings suggest that a single component or recombinant protein is not sufficient to induce an intense and sustainable Th2 response through immune-modulatory regulation and also posted risk of triggering the allergy.

Conventional administration of eggs or mix-antigen is generally subcutaneous injection, which is inevitable to high levels of invasion and pain. Fortunately, Microneedle patches, consisting of an array of micron-sized needles and a base layer, can easily pierce the stratum corneum with a thumb press or an applicator to deliver macro monomolecules into the skin [77]. Such minimally invasive and painless delivery procedure would significantly improve patients’ compliance by providing a convenient and effortless way [78]. Aiming to concurrently avoid live worm infection, reduce allergy risk and deliver egg components locally and safely to induce an individually effective immune response, we design a novel and safe delivery system, STAMP to facilitate the parasites-based T1DM therapy. With rational length optimization, the microneedles can only penetrate the epidermal layer of the skin without affecting the dermis, thus ensuring the regional delivery of inactivated eggs of *Schistosoma japonicum*. In addition, we designed an asymmetric notch at the base of the microneedle, which allows the microneedle patch effortlessly to break and thus guarantees the stable and lasting egg delivery, without a high risk of patch loss and subcutaneous contamination. It is remarkable that the immune response conducted by eggs sustained locally stimulates DCs to present antigen and maintains for at least 2 weeks, which reduces the administrative frequency and improves convenience and compliance of patients. Expectedly, it was found that STAMP could significantly suppress Th1 response and transform it to Th2 response in T1DM immunopathological changes, and simultaneously alleviate pancreatic lesions and control blood glucose in T1DM murine models. Different from others, this impact was independent of adjuvants, suggesting that the complex composition of *Schistosoma japonicum* eggs itself was sufficient to induce intense sensitization acting [79, 80]. This was possibly attributed to the fact that the microneedle system could serve as the adjuvant to prolong the persistence time of antigens in local tissues, reduce the decomposition rate of antigens, and slowly release antigens into the lymphatic system, and continuously stimulate the body to produce immune response [81]. Besides, in the STAMP system, intact eggs rather than SEA are employed, which contained a core-shell structure, thus decompose at a relatively slow rate, and effectively remaining locally on the epidermis of the skin to continuously release stimuli and generate intense immune responses, which has been proven by previous studies [53, 54]. Compared with the control group, there was no apparent alteration in body weight, life span, blood biochemistry and histological structure of main organs in T1DM mice, indicating a safe treatment by STAMP. In addition, the components of undegraded eggs will eventually be discharged as the epidermis layer is metabolized and shed, without the high risk of remaining in the skin. In this regard, it is believed that our design based on the STAMP innovated the approach of applying parasite-products to autoimmune disorders, and became an effective supplement to external insulin in the T1DM treatment.

## Conclusion

In summary, parasitic infections are a common public health problem in all over the world, but their potential protection against autoimmune and allergic diseases has been evidently observed. In order to effectively and conveniently deliver schistosome eggs to treat T1DM (a typical autoimmune disease), the STAMP system was created to provide a painless, non-invasive, controllable and safe manner, significantly improving patient compliance and adherence. Impressively, the STAMP system combines the advanced biological and engineering technologies, exhibiting both the advantages of natural and artificial species by offering long-term subcutaneous release and intense immune response (Th1 to Th2 shift). It is believed that this technology opens a new window in immunotherapy and may be applied in other autoimmune diseases in the near future.

## Methods

### Animal infection and egg purification

Schistosome eggs were collected from rabbit livers infected with *Schistosoma japonicum* (800–1000 cercariae per rabbit). Fifty days after *Schistosoma japonicum* cercariae exposure, infected rabbits were euthanized, and their livers were collected, smashed and rinsed with 1.2% sodium chloride solution which could inhibit the hatching of cercariae. Liver homogenate was trypsinized for 5 h on the incubator shaker (200 rpm, 37 ℃). The homogenate was washed with 1.2% sodium chloride solution, and filtered by nylon nets. Finally, the relatively pure homogenate was centrifuged, and the precipitate was frozen at − 80 °C. All the above operations were performed under aseptic conditions. The refrigerant *Schistosoma japonicum* eggs were lyophilized into powder after being frozen for a week and then sterilized by UV.

### Fabrication of asymmetric microneedles and STAMP

A microneedle master mold was manufactured by a high precision red wax equipment (Xiaoyanger 3D, Shenzhen, China) using a 3D printing process. Microneedles were conically shaped, with a height of 0.9 mm and a diameter of 0.5 mm; each array contained 25 microneedles. In the junction of the tips and the base layer of each microneedle, there was a cambered imperfection with a depth of 0.23 mm and a length of 0.27 mm. Poly-dimethyl-siloxane (PDMS, SYLGARD 184, Korea) was mixed with a curing agent in a 10:1 ratio, poured into a master structure, and cured at 60 °C to produce a PDMS mold. Carboxymethylcellulose (CMC, Aladdin, Shanghai, China) was dissolved in double distilled water to obtain a CMC solution with a concentration of 10% followed by crosslinking by using citric acid (CA) according to the previous method [51]. Then 500 µL of the phosphate buffered solution containing 500 µg lyophilized powder of *Schistosoma japonicum* egg or bovine serum albumin (BSA, A7906, Sigma) was loaded into PDMS mold. Centrifuged the mold for 10 min at a rotation speed of 2500 rpm to disperse the lyophilized powder evenly into each cavity. Blank microneedles without eggs (or BSA) were set as control groups. Moreover, 500 µg aluminum hydroxide microparticles (Al) were added into the mold to evaluate the adjuvant effect. After centrifugation (2500 rpm, 10 min) and vacuum (0.4 MPa, 10 min), the residual solvent of phosphate buffered solution was removed by an additional drying process at 45 °C for 1 h to obtain the dried tips containing lyophilized powder. Around 500 µl 10% CA-CMC was added to fill the 25 microneedle cavities in the PDMS mold followed by placing the mold in a vacuum chamber (JINGHONG, Shanghai, China) (0.4 MPa, 10 min). Then the mold was placed in a low-speed centrifuge (ZONKIA, Anhui, China) and centrifuged at 2500 rpm for 10 min. Repeat the preceding procedure (from vacuumizing to centrifuging) three times in total to ensure that the cavities of the mold were fully filled with CA-CMC. Place the molds in the electric thermostatic drying oven (60 ℃, 1 h). When the solution surface was concave downward half height, fill up with 10% CA-CMC. The vacuumizing to centrifuging process was conducted three times to obtain an even-distributed load of CA-CMC, followed by another drying procedure in the oven (60 ℃, 1 h). Finally, the patches loaded with different materials were obtained *via* removing them carefully from the PDMS mold after 24 h air-drying at 25 ℃. Due to the hygroscopicity of microneedles, the demolded microneedles need to be sealed in an aluminum-plastic bag or stored in a drying cabinet. The morphology of the microneedle arrays was observed on a stereomicroscope (Mshot MD50, Guangzhou, China) and a scanning electron microscope (SEM, TESCAN VEGA3 SBH/SBU, Czech).

### Mechanical strength test

The mechanical strength test was performed on an MTS 30 G tensile testing machine. With an initial spacing of 2 mm between the tips of the microneedle and the steel plate, a vertical force up to 10 N at a rate of 1 mm/min was applied to the microneedle arrays loaded with or without *Schistosoma japonicum* eggs. The mechanism properties were shown in the force-displacement patterns and the value of force was recorded when the tips began to bend.

### Skin penetration efficiency test

Microneedles loaded with Trypan blue dye, were inserted into the porcine skin. The tips were separated from the imperfection at the junction between the microneedle and the base by a transverse shear force. The constructional material CA-CMC degraded and the microchannels were stained by the Trypan blue dye released from the tips. The skin samples were photographed by the stereomicroscope.

### Fluorescence staining of *Schistosoma japonicum* eggs

500 mg lyophilized powder of *Schistosoma japonicum* egg was added to 2 ml bicarbonate buffer (0.25 M, pH = 9.8). 100 µl DMSO with 9 mg fluorescein isothiocyanate (FITC) dissolved in was added to the egg suspension. The reaction solution was stirred slowly at 4 °C overnight. Fluorescent-labeled eggs were dialyzed exhaustively (8,000 Da molecular weight cutoff) against bicarbonate buffer (0.25 M, pH = 9.8). Finally, the FITC marked egg was observed under a fluorescence microscope.

### Animal models

30 male BALB/c mice (6–8 weeks old) weighing 20–25 g were purchased from the experimental animal center of China Three Gorges University. The mice were housed in box cages, maintained on a 12-h light/12-h dark cycle, and fed with a chow diet ad libitum. Animals were randomly divided into the diabetic group and control group after an adaptation period of one week. To induce type 1 diabetes, mice of the diabetic group were treated with streptozotocin (STZ, 40 mg/kg, BioFRoxx, GRE) prepared in 0.1 M citrate buffer (pH 4.5) intraperitoneally after 6-h fasting for 5 consecutive days [82], and control group mice received an equivalent volume of citrate buffer only. The current protocol (used in our experiment) employs multiple administrations of low-dose STZ (MLD-STZ) to induce T1DM mice, which is becoming a more and more conventional and popular method for its pathogenic characteristics of chronic pancreatic islet inflammation, insulitis and insulin deficiency resembling human T1DM [82–84], and numerous studies have proved that immune responses, especially T cell-dependent immune reactions are involved and play a critical role in the pathogenesis of this model [85–90]. Tail vein fasting blood glucose (FBG) was verified weekly by glucometer (Ascensia, USA) after 6-h fasting. Mice were considered diabetes model if blood glucose concentrations increased to above 16.7 mmol/L after STZ injection and remained elevated. Following treatments, mice were euthanized, and blood and tissues were sampled for further studies. All experimental procedures were performed in accordance with animal protocols approved by the Animal Care Committee of Huazhong University of Science and Technology.

### In vivo studies using T1DM mice

Diabetes mice with ad libitum access to normal food and water on a 12 h light/dark circle was divided into four groups randomly. After all mice were fasted for 6 h, the plasma glucose concentration was determined by using a glucose meter (Baiankang, Germany) in tail vein blood samples. 6 mice each group were administered with blank MN, MN + BSA + Al, STAMP or STAMP + Al. 6 healthy mice were set as the control group and treated with blank MN. The amount of eggs, BSA and Al were set to 500 µg for each mouse. The plasma glucose concentration was monitored on d0, d7, d14 and d21. After measuring the blood glucose at the fourth week, all mice were treated for the second time with the same administration. Similarly, the blood glucose was monitored on d28, d35 and d42 after this treatment. The body weight was measured during the blood glucose detection.

### Cytokine measurement

The serum was harvested by gaining the blood from the retro-orbital plexus of mice on d14, d21 and d35 to measure the protein levels of IL-2, IFN-γ, IL-4 and IL-5. They were tested by using the specific ELISA Kits (Biosharp, China) according to the manufacturer’s protocols. All samples were measured at 480 nm in duplicate by using the TECAN Infinite 200 Pro enzyme meter.

### RNA extraction and quantitative real-time PCR

The splenocytes were collected on d42, followed by total RNA isolation by using TRIzol (Servicebio, Wuhan). Then RNA was converted into cDNA using Revert Aid First Strand cDNA Synthesis Kit (The Thermo Scientific, USA)based on the manufacturer′s instructions. The qRT-PCR assay was performed on a BIO-RAD icycler thermal cycler (Bio-Rad, USA) using SYBR Green Master Mix (biosharp, China), and the gene expression levels of T-bet, gata-3, and GAPDH were measured. Glyceraldehyde-3-phosphate dehydrogenase (GAPDH) served as the internal control. All gene primers were synthesized by Beijing Qingke Biotechnology. ΔΔCt method was used for data analysis. Total primers are described as follows:

GAPDH Forward: 5’- ACCACAGTCCATGCCATCAC-3’.

GAPDH Reverse: 5’- TCCACCACCCTGTTGCTGTA-3’.

T-bet Forward: 5’- GCCAGGGAACCGCTTATATG-3’.

T-bet Reverse: 5’- GACGATCATCTGGGTCACATTGT-3’.

Gata-3 Forward: 5’- GAGGTGGACGTACTTTTTAACATCG-3’.

Gata-3 Reverse: 5’- GGCATACCTGGCTCCCGT-3’.

### Flow cytometric analysis of Th1 and Th2 in splenocytes

The spleens were gained on d42 and mononuclear cells were extracted from the spleen of mice using 75-µm cell strainers. Then the red blood cells in samples were cracked with Red Cell Lysis Buffer (biosharp, China), followed by washing three times. After counting, the cells were cultured in RPMI 1640 medium with 10% FBS at a concentration of 1.0 × 10^7^ cells/well. Next, the cells were stimulated in a 5% CO_2_ incubator at 37 ℃ for 5 h by using Cell Activation Cocktail (without Brefeldin A) (Biolenged, USA) and Brefeldin A Solution (1000X) (Biolenged, USA) under the guidance of the manufacturer′s instructions. After twice washing, cells were stained with APC/cy7-conjugated Zombie NIR™ (Biolenged, USA) to exclude the dead cells. Regarding surface marker analysis, cells were labeled with percp/cy5.5-conjugated anti-mouse CD3ε and FITC-conjugated anti-mouse CD4^+^. Then cells were washed again, followed by the fixing and permeabilizing operation. Regarding intracellular cytokine marker analysis, cells were stained with BV-conjugated anti-mouse IL-4 and APC-conjugated anti-mouse IFN-γ. The amount of each antibody was added according to the manufacturer′s protocols. Then the cells were washed, followed by the resuspension and staining process. Next, cells were examined by a FACSCalibur flow cytometer (BECKMAN COULTER Cytoflex S, USA) and analyzed with FlowJo10.6.

### Histological analyses

The pancreas was fixed with 10% neutral buffered-formalin for 24 h and embedded in paraffin. 4-µm sections were produced and 5 sections each mouse with a 120-µm gap for each pancreas were evaluated. After being stained with hematoxylin and eosin, the sections were observed at identical exposure conditions. According to Alexandra E Livanos [91], the insulitis on a 0–4 scale was evaluated. Two blinded researchers evaluated these tissue slices and performed the insulitis scoring independently.

### Statistical analysis

All results were represented as mean ± S.D. and all statistical analyses were acted with GraphPad Prism 8.0. The significant differences between two groups were analyzed by using either Student’s *t*-test or one-way analysis of variance (ANOVA). The *p* value < 0.05 was considered statistically significant.

## Supplementary Information

**Additional file 1: Fig. S1.** Upper panels: stereomicroscopic images of regular microneedles without lyophilized *Schistosoma japonicum* eggs before (top left) and after (top right) treatment. Lower panels: regular microneedles loaded with eggs before (bottom left) and after (bottom right) treatment. **Fig. S2.** Mechanical strength test of the conventional microneedle. The tips would be ruptured when the applied force is above 0.15 N (purple arrow). **Fig. S3.** Stereomicroscopic images of microneedle tip dissolving in the sepharose gel at 0, 8 h, 16 h and 24 h. Scale bar, 1 mm. **Fig.S4. **Images of *Schistosoma japonicum* eggs under the fluorescence microscope. The eggs indicated no spontaneous fluorescence. **Fig. S5.** Blood glucose concentration vs. time curves of different treatments in the preliminary experiment. Data were presented as mean ± S.D. (n = 3). *p < 0.05, **p < 0.01, compared with the mice in the MN (T1DM) group (two-tailed Student’s *t*-test). **Fig. S6.** Weight vs. time curves of different treatments. Data were presented as mean ± S.D. (n = 6). **Fig. S7.** Blood test (WBC, RBC, PLT) and blood biochemical test (ALT, AST, CREA) after *in vivo *treatments. Data were presented as mean ± S.D.(n = 3). **Fig. S8.** Histological sections (H&E staining) of main organs from the mice in different groups. Scale bar, 50 μm. **Fig. S9. **Cytokine concentration of Th1 (IFN-γ and IL-2) and Th2 (IL-4 and IL-5) detected by ELISA on d21. Data were presented as mean ± S.D. (n = 3). ns meant no significance, *p< 0.05, **p < 0.01, compared with the mice in the MN T1DM group (two-tailed Student’s *t*-test). 

## Data Availability

The authors proclaim that the main data sustaining the results of this study are available in the article and its Additional file. Extra data are available from the corresponding authors upon request.

## Abbreviations

MN: Microneedle
T1DM: Type 1 diabetes mellitus
CA-CMC: Carboxymethyl cellulose crosslinked by citric acid
Al: Aluminum hydroxide microparticles
STAMP: *Schistosoma japonicum*-egg tip-loaded asymmetric microneedle patch
MLD-STZ: Multiple administrations of low-dose STZ
BGL: Blood glucose level

## References

[1] Zhao X, Birchall JC, Coulman SA, Tatovic D, Singh RK, Wen L, Wong FS, Dayan CM, Hanna SJ (2016). Microneedle delivery of autoantigen for immunotherapy in type 1 diabetes. J Control Release..

[2] Vojislav C, Natasa R, Milica P, Slobodan A, Radivoj K, Danijela R, Sasa R (2020). Incidence trend of type 1 diabetes mellitus in Serbia. BMC Endocr Disord..

[3] Roglic G (2016). WHO global report on diabetes: a summary. Int J Noncommun Dis..

[4] Desai S, Deshmukh A (2020). Mapping of type 1 diabetes mellitus. Curr Diabetes Rev..

[5] Bluestone JA, Herold K, Eisenbarth G (2010). Genetics, pathogenesis and clinical interventions in type 1 diabetes. Nature..

[6] Roep BO (2003). The role of T-cells in the pathogenesis of type 1 diabetes: from cause to cure. Diabetologia..

[7] Bach J-F, Chatenoud L (2012). The hygiene hypothesis: an explanation for the increased frequency of insulin-dependent diabetes. Cold Spring Harb Perspect Med.

[8] Roep BO, Thomaidou S, van Tienhoven R, Zaldumbide A (2021). Type 1 diabetes mellitus as a disease of the β-cell (do not blame the immune system?). Nat Rev Endocrinol..

[9] Kobos E, Imiela J (2015). Factors affecting the level of burden of caregivers of children with type 1 diabetes. Appl Nurs Res..

[10] Commissariat PV, Harrington KR, Whitehouse AL, Miller KM, Hilliard ME, Van Name M, DeSalvo DJ, Tamborlane WV, Anderson BJ, DiMeglio LA, Laffel LM (2020). "I’m essentially his pancreas”: parent perceptions of diabetes burden and opportunities to reduce burden in the care of children < 8 years old with type 1 diabetes. Pediatr Diabetes.

[11] Al-Mutairi HF, Mohsen AM, Al-Mazidi ZM (2007). Genetics of type I diabetes. Kuwait Med J..

[12] Acharjee S, Ghosh B, Al-Dhubiab BE, Nair AB (2013). Understanding type 1 diabetes: etiology and models. Can J Diabetes.

[13] van Belle TL, Coppieters KT, von Herrath MG (2011). Type 1 diabetes: etiology, immunology, and therapeutic strategies. Physiol Rev..

[14] Tosur M, Redondo MJ, Lyons SK (2018). Adjuvant pharmacotherapies to insulin for the treatment of type 1 diabetes. Curr Diab Rep.

[15] Waldron-Lynch F, Herold KC (2011). Immunomodulatory therapy to preserve pancreatic β-cell function in type 1 diabetes. Nat Rev Drug Discov.

[16] Tarbell KV, Petit L, Zuo X, Toy P, Luo X, Mqadmi A, Yang H, Suthanthiran M, Mojsov S, Steinman RM (2007). Dendritic cell-expanded, islet-specific CD4 + CD25 + CD62L + regulatory T cells restore normoglycemia in diabetic NOD mice. J Exp Med..

[17] Tai N, Yasuda H, Xiang Y, Zhang L, Rodriguez-Pinto D, Yokono K, Sherwin R, Wong FS, Nagata M, Wen L (2011). IL-10-conditioned dendritic cells prevent autoimmune diabetes in NOD and humanized HLA-DQ8/RIP-B7.1 mice. Clin Immunol.

[18] Ziegler A-G, Bonifacio E (2021). Shortening the paths to type 1 diabetes mellitus prevention. Nat Rev Endocrinol..

[19] Rosenzwajg M, Salet R, Lorenzon R, Tchitchek N, Roux A, Bernard C, Carel J-C, Storey C, Polak M, Beltrand J (2020). Low-dose IL-2 in children with recently diagnosed type 1 diabetes: a Phase I/II randomised, double-blind, placebo-controlled, dose-finding study. Diabetologia..

[20] Nepom GT, Ehlers M, Mandrup-Poulsen T (2013). Anti-cytokine therapies in T1D: Concepts and strategies. Clin Immunol.

[21] Pihl M, Barcenilla H, Axelsson S, Chéramy M, Åkerman L, Johansson I, Ludvigsson J, Casas R (2017). GAD-specific T cells are induced by GAD-alum treatment in Type-1 diabetes patients. Clin Immunol.

[22] Roep BO, Wheeler DCS, Peakman M (2019). Antigen-based immune modulation therapy for type 1 diabetes: the era of precision medicine. Lancet Diabetes Endocrinol..

[23] Atkinson MA, Roep BO, Posgai A, Wheeler DCS, Peakman M (2019). The challenge of modulating β-cell autoimmunity in type 1 diabetes. Lancet Diabetes Endocrinol..

[24] Yazdanbakhsh M, Kremsner PG, van Ree R (2002). Allergy, parasites, and the hygiene hypothesis. Science..

[25] Bach J-F (2018). The hygiene hypothesis in autoimmunity: the role of pathogens and commensals. Nat Rev Immunol..

[26] Saunders KA, Raine T, Cooke A, Lawrence CE (2007). Inhibition of autoimmune type 1 diabetes by gastrointestinal helminth infection. Infect Immun.

[27] Lund ME, O’Brien BA, Hutchinson AT, Robinson MW, Simpson AM, Dalton JP, Donnelly S (2014). Secreted proteins from the helminth Fasciola hepatica inhibit the initiation of autoreactive T cell responses and prevent diabetes in the NOD mouse. PLoS One..

[28] Berbudi A, Ajendra J, Wardani APF, Hoerauf A, Hübner MP (2016). Parasitic helminths and their beneficial impact on type 1 and type 2 diabetes. Diabetes Metab Res Rev..

[29] Espinoza-Jiménez A, De Haro R, Terrazas LI (2017). Antigens control experimental type 1 diabetes by inducing alternatively activated macrophages. Mediators Inflamm..

[30] Tang C-L, Zou J-N, Zhang R-H, Liu Z-M, Mao C-L (2019). Helminths protect against type 1 diabetes: effects and mechanisms. Parasitol Res..

[31] Araújo MI, Hoppe BS, Medeiros M, Carvalho EM (2004). *Schistosoma mansoni* infection modulates the immune response against allergic and auto-immune diseases. Mem Inst Oswaldo Cruz.

[32] Osada Y, Kanazawa T (2010). Parasitic helminths: new weapons against immunological disorders. J Biomed Biotechnol..

[33] Kriegel MA, Sefik E, Hill JA, Wu H-J, Benoist C, Mathis D (2011). Naturally transmitted segmented filamentous bacteria segregate with diabetes protection in nonobese diabetic mice. Proc Natl Acad Sci U S A..

[34] Lau K, Benitez P, Ardissone A, Wilson TD, Collins EL, Lorca G, Li N, Sankar D, Wasserfall C, Neu J (2011). Inhibition of type 1 diabetes correlated to a Lactobacillus johnsonii N6.2-mediated Th17 bias. J Immunol.

[35] Yan K, Wang B, Zhou H, Luo Q, Shen J, Xu Y, Zhong Z (2020). Amelioration of type 1 diabetes by recombinant fructose-1,6-bisphosphate aldolase and cystatin derived from *Schistosoma japonicum *in a murine model. Parasitol Res.

[36] Hung J-T, Liao J-H, Lin Y-C, Chang H-Y, Wu S-F, Chang T-H, Kung JT, Hsieh S-L, McDevitt H (2005). Sytwu H-K. Immunopathogenic role of TH1 cells in autoimmune diabetes: evidence from a T1 and T2 doubly transgenic non-obese diabetic mouse model. J Autoimmun.

[37] Zaccone P, Burton OT, Gibbs S, Miller N, Jones FM, Dunne DW, Cooke A (2010). Immune modulation by *Schistosoma mansoni* antigens in NOD mice: effects on both innate and adaptive immune systems. J Biomed Biotechnol..

[38] Duan Q, Xiong L, Liao C, Liu Z, Xiao Y, Huang R, Tan T, Ouyang Y, Cai J, Xiao M (2018). Population based and animal study on the effects of Schistosoma japonicum infection in the regulation of host glucose homeostasis. Acta Trop..

[39] Chen W, Wainer J, Ryoo SW, Qi X, Chang R, Li J, Lee SH, Min S, Wentworth A, Collins JE (2022). Dynamic omnidirectional adhesive microneedle system for oral macromolecular drug delivery. Sci Adv..

[40] Chen W, Wang Z, Wang L, Chen X (2022). Smart chemical engineering-based lightweight and miniaturized attachable systems for advanced drug delivery and diagnostics. Adv Mater..

[41] Chen W, Cai B, Geng Z, Chen F, Wang Z, Wang L, Chen X (2020). Reducing false negatives in COVID-19 testing by using microneedle-based oropharyngeal swabs. Matter..

[42] Cai B, Gong Y, Wang Z, Wang L, Chen W (2021). Microneedle arrays integrated with living organisms for smart biomedical applications. Theranostics.

[43] Chen W, Tian R, Xu C, Yung BC, Wang G, Liu Y, Ni Q, Zhang F, Zhou Z, Wang J (2017). Microneedle-array patches loaded with dual mineralized protein/peptide particles for type 2 diabetes therapy. Nat Commun..

[44] Kim Y-C, Park J-H, Prausnitz MR (2012). Microneedles for drug and vaccine delivery. Adv Drug Deliv Rev..

[45] Haq MI, Smith E, John DN, Kalavala M, Edwards C, Anstey A, Morrissey A, Birchall JC (2009). Clinical administration of microneedles: skin puncture, pain and sensation. Biomed Microdevices.

[46] Zhang Y, Yu J, Kahkoska AR, Wang J, Buse JB, Gu Z (2019). Advances in transdermal insulin delivery. Adv Drug Deliv Rev.

[47] Wang P, Wang Y, Yi Y, Gong Y, Ji H, Gan Y, Xie F, Fan J, Wang X (2022). MXenes-integrated microneedle combined with asiaticoside to penetrate the cuticle for treatment of diabetic foot ulcer. J Nanobiotechnology..

[48] Demuth PC, Garcia-Beltran WF, Ai-Ling ML, Hammond PT, Irvine DJ (2013). Composite dissolving microneedles for coordinated control of antigen and adjuvant delivery kinetics in transcutaneous vaccination. Adv Funct Mater..

[49] Amani H, Shahbazi M-A, D’Amico C, Fontana F, Abbaszadeh S, Santos HA (2021). Microneedles for painless transdermal immunotherapeutic applications. J Control Release..

[50] Park J, Kim Y-C (2021). Topical delivery of 5-fluorouracil-loaded carboxymethyl chitosan nanoparticles using microneedles for keloid treatment. Drug Deliv Transl Res.

[51] Capanema NSV, Mansur AAP, de Jesus AC, Carvalho SM, de Oliveira LC, Mansur HS (2018). Superabsorbent crosslinked carboxymethyl cellulose-PEG hydrogels for potential wound dressing applications. Int J Biol Macromol.

[52] Kim JD, Kim M, Yang H, Lee K, Jung H (2013). Droplet-born air blowing: novel dissolving microneedle fabrication. J Control Release..

[53] Candido RRF, Favero V, Duke M, Karl S, Gutiérrez L, Woodward RC, Graeff-Teixeira C, Jones MK, St Pierre TG (2015). The affinity of magnetic microspheres for Schistosoma eggs. Int J Parasitol..

[54] Karl S, Gutiérrez L, Lucyk-Maurer R, Kerr R, Candido RRF, Toh SQ, Saunders M, Shaw JA, Suvorova A, Hofmann A (2013). The iron distribution and magnetic properties of schistosome eggshells: implications for improved diagnostics. PLoS Negl Trop Dis..

[55] Strachan DP (1989). Hay fever, hygiene, and household size. BMJ.

[56] Weintrob N, Sprecher E, Israel S, Pinhas-Hamiel O, Kwon OJ, Bloch K, Abramov N, Arbel A, Josefsberg Z, Brautbar C, Vardi P (2001). Type 1 diabetes environmental factors and correspondence analysis of HLA class II genes in the Yemenite Jewish community in Israel. Diabetes Care..

[57] Elliott DE, Weinstock JV (2012). Helminth-host immunological interactions: prevention and control of immune-mediated diseases. Ann N Y Acad Sci..

[58] Du L, Tang H, Ma Z, Xu J, Gao W, Chen J, Gan W, Zhang Z, Yu X, Zhou X, Hu X (2011). The protective effect of the recombinant 53-kDa protein of Trichinella spiralis on experimental colitis in mice. Dig Dis Sci..

[59] Weinstock JV, Elliott DE (2013). Translatability of helminth therapy in inflammatory bowel diseases. Int J Parasitol..

[60] Leonardi I, Gerstgrasser A, Schmidt TSB, Nicholls F, Tewes B, Greinwald R, von Mering C, Rogler G, Frey-Wagner I (2017). Preventive Trichuris suis ova (TSO) treatment protects immunocompetent rabbits from DSS colitis but may be detrimental under conditions of immunosuppression. Sci Rep.

[61] Kron MA, Metwali A, Vodanovic-Jankovic S, Elliott D (2013). Nematode asparaginyl-tRNA synthetase resolves intestinal inflammation in mice with T-cell transfer colitis. Clin Vaccine Immunol.

[62] Navarro S, Pickering DA, Ferreira IB, Jones L, Ryan S, Troy S, Leech A, Hotez PJ, Zhan B, Laha T (2016). Hookworm recombinant protein promotes regulatory T cell responses that suppress experimental asthma. Sci Transl Med..

[63] Hirahara K, Vahedi G, Ghoreschi K, Yang X-P, Nakayamada S, Kanno Y, O’Shea JJ, Laurence A (2011). Helper T-cell differentiation and plasticity: insights from epigenetics. Immunology..

[64] Martínez-Méndez D, Villarreal C, Mendoza L, Huerta L (2020). An integrative network modeling approach to T CD4 cell activation. Front Physiol.

[65] McClymont SA, Putnam AL, Lee MR, Esensten JH, Liu W, Hulme MA, Hoffmüller U, Baron U, Olek S, Bluestone JA, Brusko TM (2011). Plasticity of human regulatory T cells in healthy subjects and patients with type 1 diabetes. J Immunol..

[66] McSorley HJ, Maizels RM (2012). Helminth infections and host immune regulation. Clin Microbiol Rev..

[67] Fairfax K, Nascimento M, Huang SC-C, Everts B, Pearce EJ (2012). Th2 responses in schistosomiasis. Semin Immunopathol.

[68] Cooke A, Tonks P, Jones FM, O’Shea H, Hutchings P, Fulford AJ, Dunne DW (1999). Infection with *Schistosoma mansoni *prevents insulin dependent diabetes mellitus in non-obese diabetic mice. Parasite Immunol.

[69] Zaccone P, Burton O, Miller N, Jones FM, Dunne DW, Cooke A (2009). Schistosoma mansoni egg antigens induce Treg that participate in diabetes prevention in NOD mice. Eur J Immunol.

[70] Cleenewerk L, Garssen J, Hogenkamp A (2020). Clinical use of antigens as novel immunotherapies for autoimmune disorders. Front Immunol.

[71] Zaccone P, Fehérvári Z, Jones FM, Sidobre S, Kronenberg M, Dunne DW, Cooke A (2003). Schistosoma mansoni antigens modulate the activity of the innate immune response and prevent onset of type 1 diabetes. Eur J Immunol..

[72] Zaccone P, Burton OT, Gibbs SE, Miller N, Jones FM, Schramm G, Haas H, Doenhoff MJ, Dunne DW, Cooke A (2011). The S. mansoni glycoprotein ω-1 induces Foxp3 expression in NOD mouse CD4+ T cells. Eur J Immunol..

[73] Zou J, Liu W, Lei J, Mo H, Wang C, Yu G, Cheng Y, Li Y (2006). Effect of chronic infection with *Schistosoma japonicum *on multiple low-dose streptozotocin induced diabetes mellitus in mice. J Pathog Biol..

[74] Hams E, Aviello G, Fallon PG (2013). The schistosoma granuloma: friend or foe?. Front Immunol.

[75] Wang L, Yu Z, Wan S, Wu F, Chen W, Zhang B, Lin D, Liu J, Xie H, Sun X, Wu Z (2017). Exosomes derived from dendritic cells treated with soluble egg antigen attenuate DSS-induced colitis. Front Pharmacol.

[76] Wang S, Xie Y, Yang X, Wang X, Yan K, Zhong Z, Wang X, Xu Y, Zhang Y, Liu F, Shen J (2016). Therapeutic potential of recombinant cystatin from *Schistosoma japonicum* in TNBS-induced experimental colitis of mice. Parasit Vectors..

[77] McCrudden MTC, McAlister E, Courtenay AJ, González-Vázquez P, Singh TRR, Donnelly RF (2015). Microneedle applications in improving skin appearance. Exp Dermatol..

[78] Richter-Johnson J, Kumar P, Choonara YE, du Toit LC, Pillay V (2018). Therapeutic applications and pharmacoeconomics of microneedle technology. Expert Rev Pharmacoecon Outcomes Res..

[79] Ohta N, Asahi H, Hosaka Y, Minai M, Ishii A (1991). Regulation of the human T-cell response to *Schistosoma japonicum* egg antigen by concomitant cellular and humoral mechanisms in vitro. Parasitol Res.

[80] Carson JP, Robinson MW, Hsieh MH, Cody J, Le L, You H, McManus DP, Gobert GN (2020). A comparative proteomics analysis of the egg secretions of three major schistosome species. Mol Biochem Parasitol..

[81] He P, Zou Y, Hu Z (2015). Advances in aluminum hydroxide-based adjuvant research and its mechanism. Hum Vaccin Immunother.

[82] Furman BL (2015). Streptozotocin-induced diabetic models in mice and rats. Curr Protocols Pharmacol..

[83] Like AA, Rossini AA (1976). Streptozotocin-induced pancreatic insulitis: new model of diabetes mellitus. Science.

[84] Kolb-Bachofen V, Epstein S, Kiesel U, Kolb H (1988). Low-dose streptozocin-induced diabetes in mice. Electron microscopy reveals single-cell insulitis before diabetes onset. Diabetes.

[85] Paik SG, Fleischer N, Shin SI (1980). Insulin-dependent diabetes mellitus induced by subdiabetogenic doses of streptozotocin: obligatory role of cell-mediated autoimmune processes. Proc Natl Acad Sci U S A..

[86] Herold KC, Montag AG, Fitch FW (1987). Treatment with anti-T-lymphocyte antibodies prevents induction of insulitis in mice given multiple doses of streptozocin. Diabetes..

[87] Klinkhammer C, Popowa P, Gleichmann H (1988). Specific immunity to streptozocin. Cellular requirements for induction of lymphoproliferation. Diabetes.

[88] Klinkhammer C, Dohle C, Gleichmann H (1989). T cell-dependent class II major histocompatibility complex antigen expression in vivo induced by the diabetogen streptozotocin. Immunobiology..

[89] Cockfield SM, Ramassar V, Urmson J, Halloran PF (1989). Multiple low dose streptozotocin induces systemic MHC expression in mice by triggering T cells to release IFN-gamma. J Immunol.

[90] Lukić ML, Stosić-Grujicić S, Shahin A (1998). Effector mechanisms in low-dose streptozotocin-induced diabetes. Dev Immunol.

[91] Livanos AE, Greiner TU, Vangay P, Pathmasiri W, Stewart D, McRitchie S, Li H, Chung J, Sohn J, Kim S (2016). Antibiotic-mediated gut microbiome perturbation accelerates development of type 1 diabetes in mice. Nat Microbiol..

