# Supplementary material for: Immunotherapy for type 1 diabetes mellitus by adjuvant-free Schistosoma japonicum-egg tip-loaded asymmetric microneedle patch (STAMP)
Source: J Nanobiotechnology. 2022 Aug 13;20:377. doi: 10.1186/s12951-022-01581-9 (PMC9375265; doi:10.1186/s12951-022-01581-9)
Supplement: Supplementary file 1 — Additional file 1: Fig. S1. Upper panels: stereomicroscopic images of regular microneedles without lyophilized Schistosoma japonicum eggs before (top left) and after (top right) treatment. Lower panels: regular microneedles loaded with eggs before (bottom left) and after (bottom right) treatment. Fig. S2. Mechanical strength test of the conventional microneedle. The tips would be ruptured when the applied force is above 0.15 N (purple arrow). Fig. S3. Stereomicroscopic images of microneedle tip dissolving in the sepharose gel at 0, 8 h, 16 h and 24 h. Scale bar, 1 mm. Fig.S4. Images of Schistosoma japonicum eggs under the fluorescence microscope. The eggs indicated no spontaneous fluorescence. Fig. S5. Blood glucose concentration vs. time curves of different treatments in the preliminary experiment. Data were presented as mean ± S.D. (n = 3). *p < 0.05, **p < 0.01, compared with the mice in the MN (T1DM) group (two-tailed Student’s t-test). Fig. S6. Weight vs. time curves of different treatments. Data were presented as mean ± S.D. (n = 6). Fig. S7. Blood test (WBC, RBC, PLT) and blood biochemical test (ALT, AST, CREA) after in vivo treatments. Data were presented as mean ± S.D.(n = 3). Fig. S8. Histological sections (H&E staining) of main organs from the mice in different groups. Scale bar, 50 μm. Fig. S9. Cytokine concentration of Th1 (IFN-γ and IL-2) and Th2 (IL-4 and IL-5) detected by ELISA on d21. Data were presented as mean ± S.D. (n = 3). ns meant no significance, *p< 0.05, **p < 0.01, compared with the mice in the MN T1DM group (two-tailed Student’s t-test). [file 12951_2022_1581_MOESM1_ESM.docx]

**Additional file 1**

**Immunotherapy for Type 1 Diabetes Mellitus by Adjuvant-free *Schistosoma japonicum*-eggs Tip-loaded** **Asymmetric Microneedle Patch (STAMP)**

Haoming Huang^1,^^†^, Dian Hu^1^^,†^, Zhuo Chen^1,†^, Jiarong Xu^2^, Rengui Xu^2^, Yusheng Gong^2^, Zhengming Fang^3^, Ting Wang^1,3*^ and Wei Chen^1,2,4*^

^†^ Haoming Huang, Dian Hu and Zhuo Chen contributed to this work equally.

^1^National Demonstration Center for Experimental Basic Medical Education, School of Basic Medicine, Tongji Medical College, Huazhong University of Science and Technology, Wuhan, Hubei 430030, China

^2^Department of Pharmacology, School of Basic Medicine, Tongji Medical College, Huazhong University of Science and Technology, Wuhan, Hubei 430030, China

^3^Department of Pathogen Biology, School of Basic Medicine, Tongji Medical College, Huazhong University of Science and Technology, Wuhan, Hubei 430030, China

^4^Hubei Key Laboratory for Drug Target Researches and Pharmacodynamic Evaluation, Huazhong University of Science and Technology, Wuhan Hubei 430030, China

^*^Correspondence: weichen86@hust.edu.cn; wangting139@hust.edu.cn

**Additional file 1 Figures & Legends**


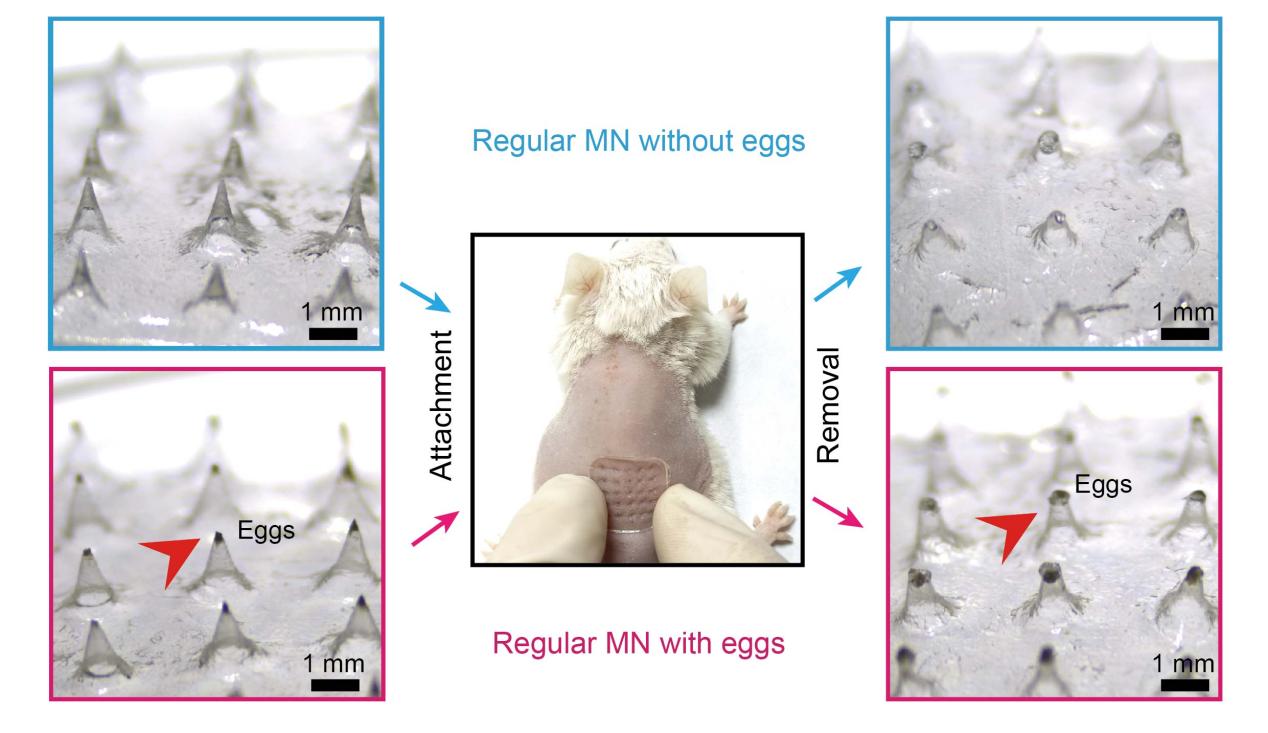


**Fig. S1.** Upper panels: stereomicroscopic images of regular microneedles without lyophilized *Schistosoma japonicum* eggs before (top left) and after (top right) treatment. Lower panels: regular microneedles loaded with eggs before (bottom left) and after (bottom right) treatment.


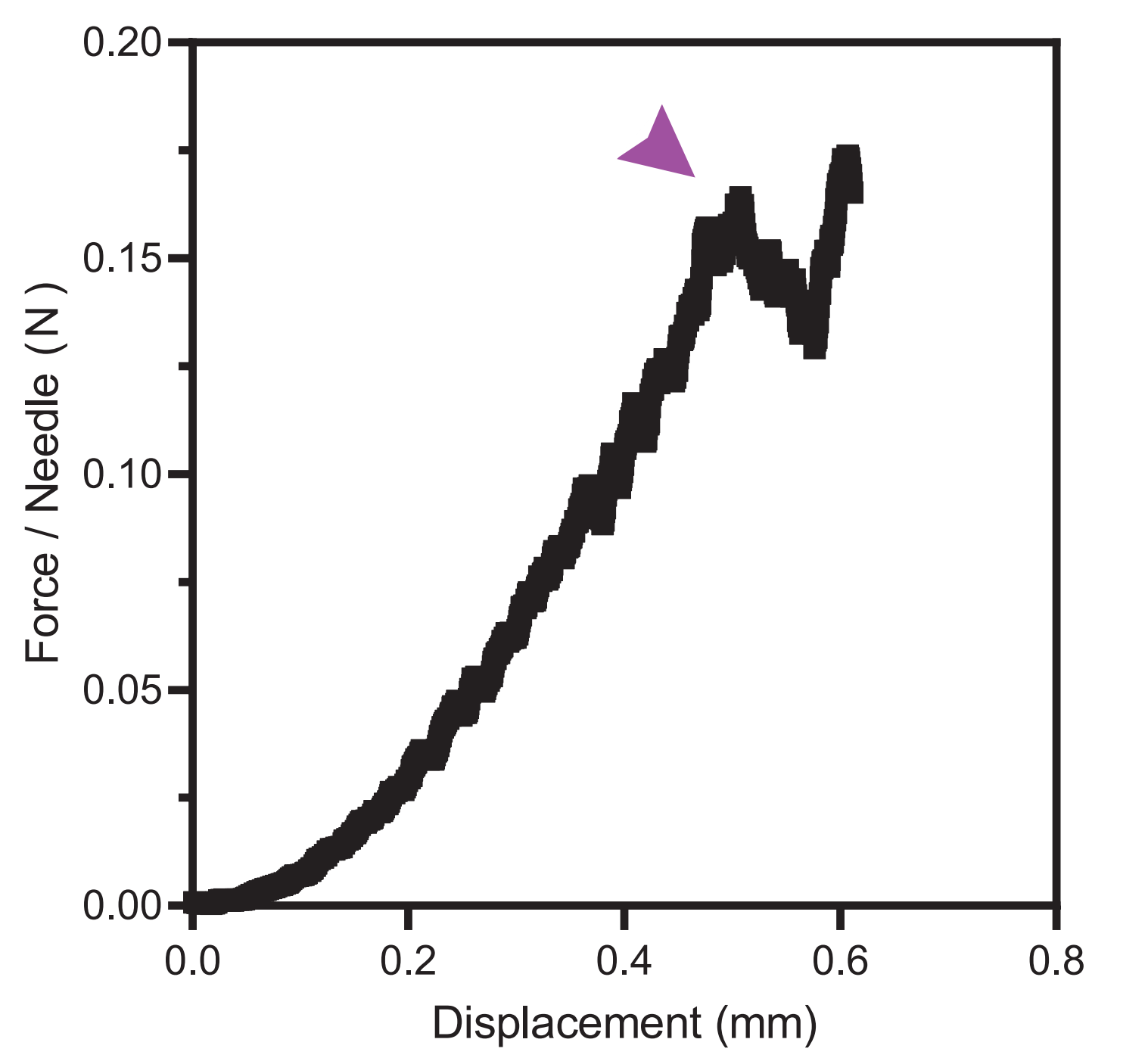


Fig. S2. Mechanical strength test of the conventional microneedle. The tips would be ruptured when the applied force is above 0.15 N (purple arrow).


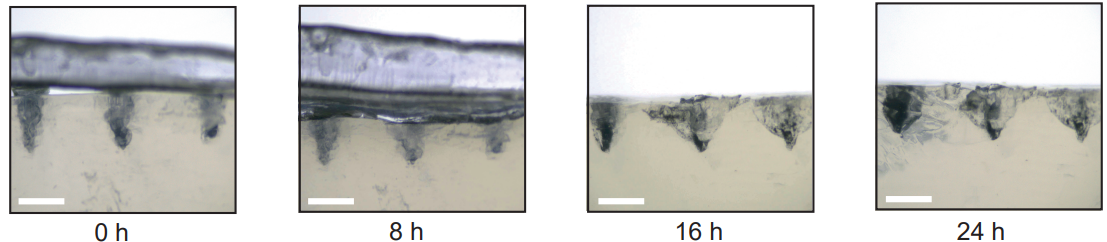


**Fig. S3.** Stereomicroscopic images of microneedle tip dissolving in the sepharose gel at 0, 8 h, 16 h and 24 h. Scale bar, 1 mm.

**
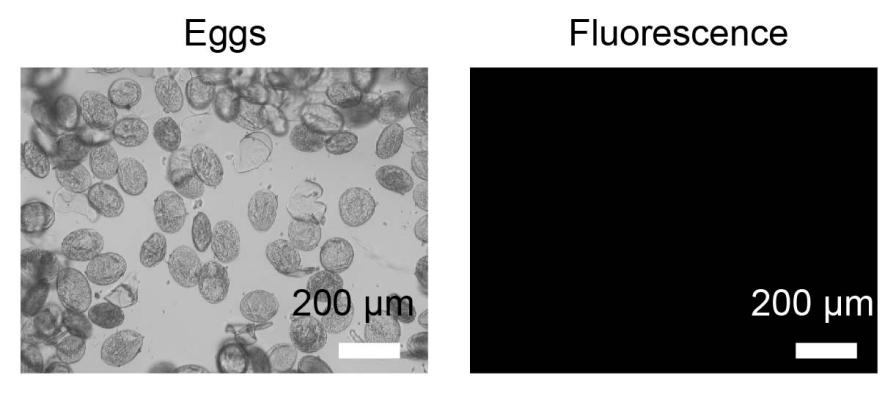
**

**Fig. S4.** Images of *Schistosoma japonicum* eggs under the fluorescence microscope. The eggs indicated no spontaneous fluorescence.


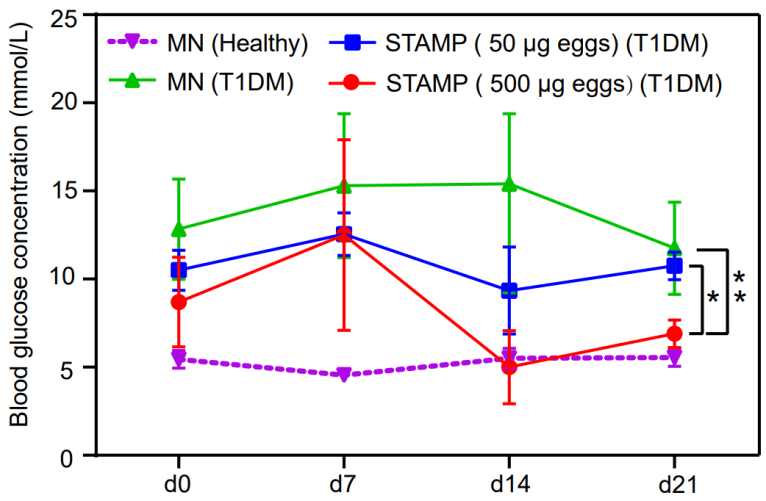


**Fig. S5.** Blood glucose concentration vs. time curves of different treatments in the preliminary experiment. Data were presented as mean ± S.D. (n = 3). **p* < 0.05, ***p* < 0.01, compared with the mice in the MN (T1DM) group (two-tailed Student’s *t*-test).

**
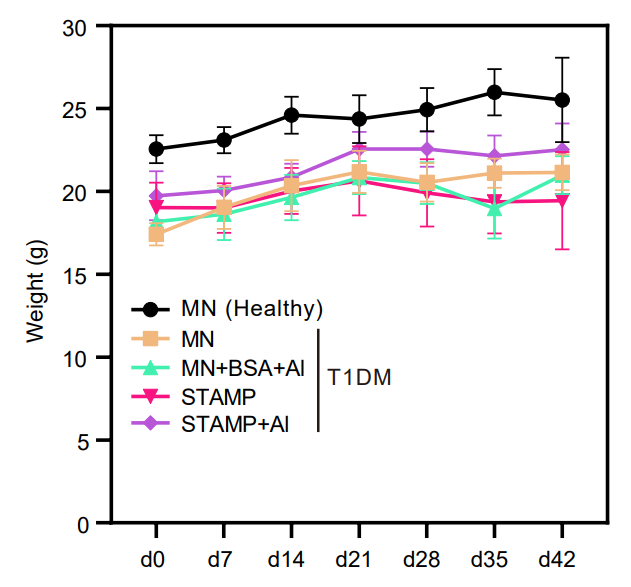
**

**Fig. S6.** Weight vs. time curves of different treatments. Data were presented as mean ± S.D. (n = 6).


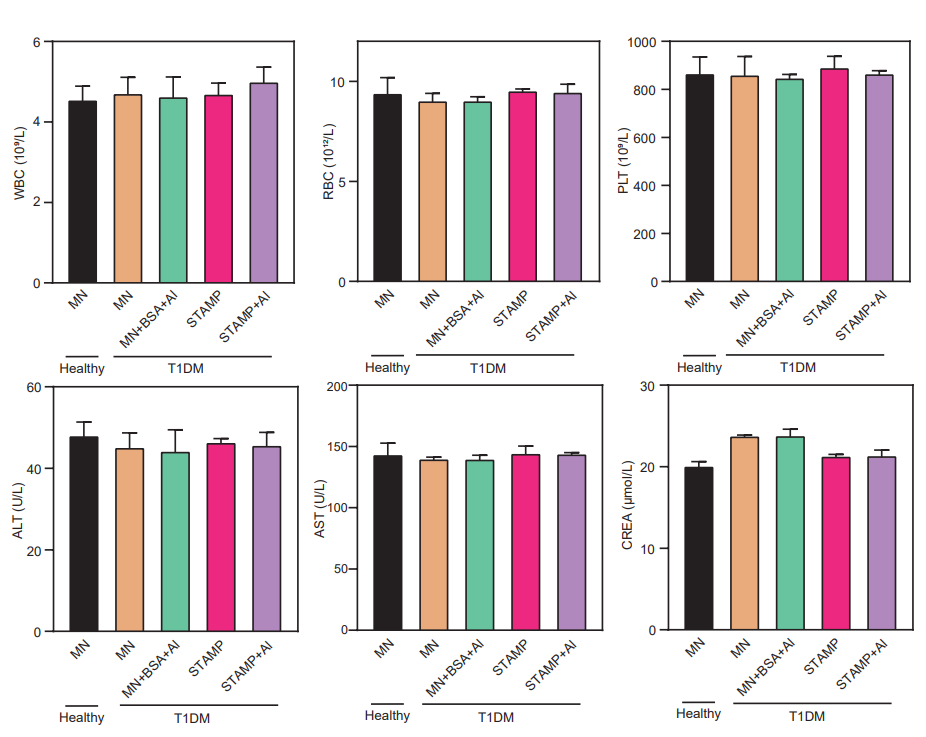


**Fig. S7.** Blood test (WBC, RBC, PLT) and blood biochemical test (ALT, AST, CREA) after *in vivo* treatments. Data were presented as mean ± S.D. (n = 3).


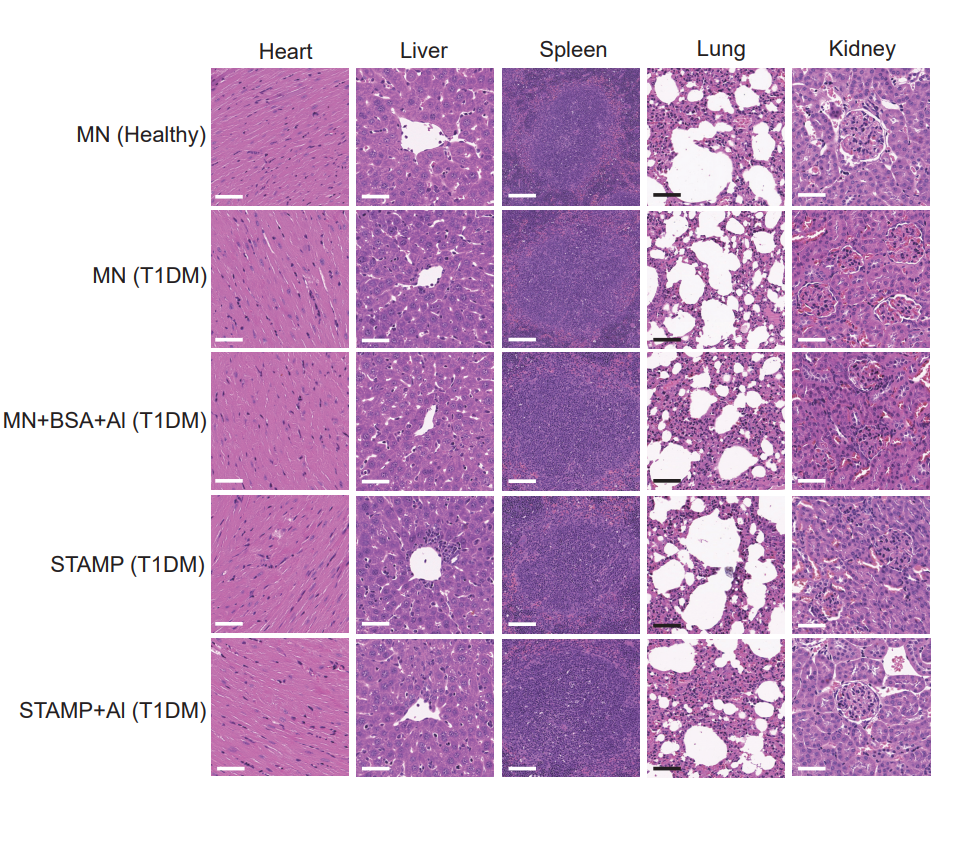


**Fig. S8.** Histological sections (H&E staining) of main organs from the mice in different groups. Scale bar, 50 μm.


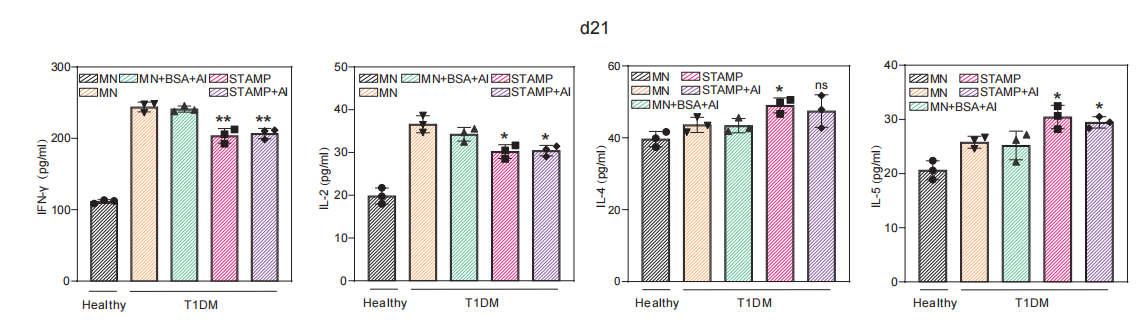


**Fig. S9.** Cytokine concentration of Th1 (IFN-γ and IL-2) and Th2 (IL-4 and IL-5) detected by ELISA on d21. Data were presented as mean ± S.D. (n = 3). ns meant no significance*,* **p* < 0.05, ***p* < 0.01, compared with the mice in the MN T1DM group (two-tailed Student’s *t*-test).
